# Activation of cannabinoid-2 receptor protects against *Pseudomonas aeruginosa* induced acute lung injury and inflammation

**DOI:** 10.1186/s12931-022-02253-w

**Published:** 2022-12-03

**Authors:** Nagaraja Nagre, Gregory Nicholson, Xiaofei Cong, Janette Lockett, Andrew C. Pearson, Vincent Chan, Woong-Ki Kim, K. Yaragudri Vinod, John D. Catravas

**Affiliations:** 1 Department of Physiological Sciences, Eastern Virginia Medical School, Norfolk, VA 23507 USA; 2 Department of Microbiology and Molecular Cell Biology, Eastern Virginia Medical School, Norfolk, VA 23507 USA; 3 Emotional Brain Institute, Nathan Kline Institute for Psychiatric Research, Orangeburg, NY 10962 USA; 4 Department of Child and Adolescent Psychiatry, New York University Langone Health, New York, NY USA; 5 Frank Reidy Research Center for Bioelectrics, Old Dominion University, Norfolk, VA 23508 USA; 6 School of Medical Diagnostic and Translational Sciences, College of Health Sciences, Old Dominion University, Norfolk, VA 23508 USA

**Keywords:** Bacterial pneumonia, *Pseudomonas aeruginosa*, Acute lung injury and inflammation, Cannabinoid-2 receptor

## Abstract

**Background:**

Bacterial pneumonia is a major risk factor for acute lung injury (ALI) and acute respiratory distress syndrome (ARDS). *Pseudomonas aeruginosa* (PA), an opportunistic pathogen with an increasing resistance acquired against multiple drugs, is one of the main causative agents of ALI and ARDS in diverse clinical settings. Given the anti-inflammatory role of the cannabinoid-2 receptor (CB2R), the effect of CB2R activation in the regulation of PA-induced ALI and inflammation was tested in a mouse model as an alternative to conventional antibiotic therapy.

**Methods:**

In order to activate CB2R, a selective synthetic agonist, JWH133, was administered intraperitoneally (i.p.) to C57BL/6J mice. Furthermore, SR144528 (a selective CB2R antagonist) was administered in combination with JWH133 to test the specificity of the CB2R-mediated effect. PA was administered intratracheally (i.t.) for induction of pneumonia in mice. At 24 h after PA exposure, lung mechanics were measured using the FlexiVent system. The total cell number, protein content, and neutrophil population in the bronchoalveolar lavage fluid (BALF) were determined. The bacterial load in the whole lung was also measured. Lung injury was evaluated by histological examination and PA-induced inflammation was assessed by measuring the levels of BALF cytokines and chemokines. Neutrophil activation (examined by immunofluorescence and immunoblot) and PA-induced inflammatory signaling (analyzed by immunoblot) were also studied.

**Results:**

CB2R activation by JWH133 was found to significantly reduce PA-induced ALI and the bacterial burden. CB2R activation also suppressed the PA-induced increase in immune cell infiltration, neutrophil population, and inflammatory cytokines. These effects were abrogated by a CB2R antagonist, SR144528, further confirming the specificity of the CB2R-mediated effects. CB2R-knock out (CB2RKO) mice had a significantly higher level of PA-induced inflammation as compared to that in WT mice. CB2R activation diminished the excess activation of neutrophils, whereas mice lacking CB2R had elevated neutrophil activation. Pharmacological activation of CB2R significantly reduced the PA-induced NF-κB and NLRP3 inflammasome activation, whereas CB2KO mice had elevated NLRP3 inflammasome.

**Conclusion:**

Our findings indicate that CB2R activation ameliorates PA-induced lung injury and inflammation, thus paving the path for new therapeutic avenues against PA pneumonia.

## Background

Bacterial pneumonia is a widespread respiratory disease. The inflammatory response initiated by bacterial pathogens can spiral out of control and lead to acute lung injury (ALI) or acute respiratory distress syndrome (ARDS) [1, 2]. ARDS is a life-threatening form of respiratory failure and is often characterized by an overwhelming, dysregulated inflammatory environment in the lung accompanied by rapid recruitment of neutrophils into the alveolar space, interstitial edema, and injury to epithelial and endothelial cells [3, 4]. One of the most common causative agents of ARDS is *Pseudomonas aeruginosa* (PA), a multidrug-resistant (MDR) opportunistic pathogen, associated with respiratory tract infections in diverse clinical settings. PA accounts for nearly 10% of hospital-acquired infections and about 17% of ventilator-associated pneumonia (VAP) with attributable mortality up to 40% [5–7]. The increasing multidrug resistance of PA has placed this bacterium on the WHO priority pathogens list for the development of new treatment options [8]. Antibiotic therapies are found to be ineffective against multidrug-resistant PA in critically ill patients, leading to increased mortality and worsening outcomes. Therefore, novel non-antibiotic therapeutic options need to be explored to counter PA infection and subsequent ARDS.

The endocannabinoid system has a remarkable distribution in diverse physiological functions of mammals, one of them being its natural mechanism to control aberrant inflammatory responses [9, 10]. This system is composed of cannabinoid (CB) receptors; endocannabinoids such as N-arachidonyl ethanolamine (anandamide/AEA) and 2-arachidonylglycerol (2-AG); and enzymes involved in their biosynthesis and degradation. These endocannabinoids (AEA and 2-AG) exert their effects mainly through binding to two G-protein coupled receptors (GPCRs): CB1R (a CB1 receptor) and CB2R respectively [11–13]. Alterations in the endocannabinoid system are found in patients with certain diseases and in experimental models. The pharmacological and genetic modifications of this system in mice models have shown alterations in their susceptibility to several conditions including neurodegenerative, cardiovascular, and gastrointestinal disorders [11, 14], pointing towards the components of the endocannabinoid system as possible therapeutic targets. While CB1R is highly expressed in the brain, CB2R is predominantly found in the peripheral system. Both these receptors exhibit an immunomodulatory role by several mechanisms including development, migration, proliferation, and effector functions [13, 15]. Activation of CB2R displays anti-inflammatory and anti-fibrogenic effects without eliciting any adverse psychotic effects. Hence, understanding the role of CB2R in immune regulation is gaining attention from a therapeutic perspective [16, 17]. Pharmacological activation of CB2R prevents nephrotoxicity and reduces inflammation, oxidative stress, and cell death in nephropathy [18, 19]. CB2R activation by selective synthetic agonists has been shown to protect against tissue damage in experimental models of ischemic-reperfusion injury [20, 21]. In the lung, pharmacological activation of CB2R was found to have a protective effect on paraquat-induced acute lung injury in a rat model and alleviate bleomycin-induced pulmonary fibrosis in a mouse model [22, 23]. However, the impact of CB2R signaling on bacterial pneumonia-induced lung injury is largely unknown.

Another notable system that is assembled in response to microbial infection and cellular stresses is inflammasomes, which are a group of cytosolic protein complexes playing a pivotal role in innate immunity and inflammation [24]. Inflammasomes consist of a nucleotide-binding oligomerization domain (NOD)-like receptor (NLR); an adapter which is an apoptosis-associated speck-like protein containing a caspase recruitment domain (ASC); and an effector protease caspase-1. The gathering and assembly of these protein complexes result in the activation of caspase-1, assisting in the maturation of the pro-inflammatory cytokines, interleukin-1β (IL-1β) and IL-18, into biologically active forms [25, 26]. A member of the NLR inflammasome family is the NLRP3 (NOD-; leucine-rich repeat, LRR-; and pyrin domain-containing protein 3) that is activated by a wide range of stimuli including pathogen-associated molecular patterns (PAMPs) and/or host-derived damage-associated molecular patterns (DAMPs) [27, 28]. Currently, a two-signal model has been proposed for NLRP3 inflammasome activation. The first priming signal comes from the stimulation of pathogen recognition receptors (PRRs) that activate the nuclear factor- κB (NF-κB) and the second signal (activation) is induced by factors like ATP, pore formation, potassium efflux, lysosomal rupture, and reactive oxygen species [29].

Activation of NLRP3 during different bacterial infections is a part of the innate immune response [30] and has been implicated in various animal models of ALI such as mechanical ventilation [31], hyperoxia [32], and polymicrobial sepsis [33]. Further, the absence of interleukin-1 (IL-1) receptor (IL-1R) or IL-18 was found to improve host defense against PA-induced inflammatory responses [34, 35], and activation of inflammasome was found to mediate pathology of acute PA-pneumonia [36]. The significance of NLRP3 activation by PA has also been recently highlighted in a mouse model [37].

In this study, we demonstrated that pharmacological activation of CB2R by a selective synthetic agonist JWH133 effectively reduced the PA-induced lung injury and inflammation. JWH133 treatment was also found to significantly reduce neutrophil infiltration and prevent the excessive activation of neutrophils. On the other hand, deficiency of CB2R aggravated PA-induced lung injury and inflammation. It was further observed that CB2R activation suppressed the activation of NF-κB and NLRP3 inflammasome, while CB2KO mice were found to have elevated levels of NLRP3 after PA infection.

## Materials and methods

### Animals

CB2KO mice were procured from the Jackson Laboratory (stock # 005876). Mice were backcrossed to C57BL/6 J mice for > 10 generations and C57BL/6 J WT mice were used as controls. Mice were housed in a sterile ventilated facility at Eastern Virginia Medical School (EVMS) under standard husbandry. Mice of mixed gender and 12–14 weeks of age were used in this study. All procedures were approved by the Institutional Animal Care and Use Committee of EVMS.

### Animal procedures

*P. aeruginosa* (strain: PA01) was a generous gift from Dr. Peter Di, University of Pittsburgh. PA01 was grown on *Pseudomonas* Isolation Agar plates (Sigma-Aldrich) for 16 h and the colonies were suspended and grown in Lysogeny Broth (LB). The concentrations of PA01 in LB were estimated by measuring the optical density at 600 nm. The PA culture was further diluted in phosphate-buffered saline (PBS) to obtain the required colony-forming units (CFU). PA pneumonia was modeled by intratracheal (i.t.) injection of PA (3 × 10^7^ CFU in 50 μl) into mice using a micro sprayer (Penn Century Inc.) under anesthesia with ketamine and xylazine. The control mice received 50 μl PBS via i.t. injections. CB2R agonist JWH133 (1738, Tocris Biosciences) and CB2R antagonist SR144528 (5039, Tocris Biosciences) were dissolved in a vehicle (1% DMSO + 1% Tween-80 in PBS). In order to activate CB2R, JWH133 (5 mg/kg) was administered to mice via the intraperitoneal (i.p.) route, 1 h prior to PA infection. The specificity of CB2R mediated effect was tested by administering an i.p. injection of SR144528 (2 mg/kg) to mice, 30 min prior to JWH133 injection. Age-matched (12–14 weeks) C57BL/6 J WT mice of mixed gender were distributed into the following groups: (1) vehicle + PBS, (2) JWH133 + PBS, (3) SR144528 + PBS, (4) vehicle + PA, (5) JWH133 + PA, (6) SR144528 + JWH133 + PA. Acute lung injury was assessed at 24 h after PA infection. In order to test the effect of genetic deletion of CB2R, CB2RKO mice were exposed to PA as described above. Acute lung injury was assessed at 24 h after PA infection in WT and CB2KO mice.

### Measurement of lung mechanics

Lung mechanics were measured using the FlexiVent system and FlexiWare software (SCIREQ, Montreal, Canada). After 24 h of PA infection, mice were anesthetized using ketamine/xylazine. Anesthetized mice were intubated intratracheally with a calibrated 18½ G catheter and ventilated at lower tidal volume (10 ml/kg) and 150 breaths/minute. Tissue elastance (H) and tissue dampening (G) and static lung compliance (Cst) were assessed using FlexiWare software, according to manufacturer recommendations.

### Analysis of bronchoalveolar lavage fluid (BALF)

After measuring the lung mechanics, mice lungs were lavaged with 3 ml of PBS and the total cell number in the BALF was determined using an automated cell counter (Countess II FL, ThermoFisher Scientific). The total protein content in the BALF was determined by BCA Protein Assay Kit (Bio-Rad Laboratories). A total of 0.5 × 10^6^ BALF cells were incubated with anti-rat-FcRII/III antibody (Fc block, BD Biosciences) for 10 min at 4 °C to block nonspecific binding. This was followed by 30 min of incubation with an antibody cocktail containing APC-anti-F4/80 (123115, BioLegend) and PE-anti-Ly6G (127607, BioLegend). Cells were washed in cold PBS and re-suspended in PBS with 2% fetal bovine serum (FBS) and analyzed on flow cytometry (Cytek Aurora). All data were analyzed using the Flow Jo software version 10.2. Levels of cytokines (IL-1β, Il-6, and TNF-α) and chemokines (KC and MIP-2) in the BALF were determined by ELISA (R&D Systems).

### Lung tissue processing

After collecting the BALF, the right mainstem bronchus was tied off with a 4–0 silk suture, and the right lung was cut and snap-frozen in liquid nitrogen and stored at − 80 °C until further use. The left lung was inflated with 4% paraformaldehyde (PFA) at 20 cm H_2_O and fixed overnight at 4 °C. The left lobe was processed for hematoxylin and eosin (H&E) staining to assess lung injury and immune cell infiltration.

### Measurement of bacterial clearance

In order to test lung bacterial burden, the whole lung was suspended in 200 μl PBS and homogenized using a previously tested setting on an electronic homogenizer (TH-01, Omini Inc.) that completely disrupted lung tissue without breaking the bacteria. The total volume was brought to 1 ml, and 100 μl of lung lysate was plated on *Pseudomonas* Isolation Agar plates (Sigma-Aldrich) at tenfold serial dilutions. Plates were incubated at 37 °C for 24 h, and bacterial colonies were counted to determine the CFU of the whole lung.

### Isolation and treatment of mouse primary alveolar macrophages (AMs)

Mouse primary AMs were isolated by lavage of the WT mouse lung using 3 ml PBS, 1 ml at a time. The collected cell suspension was centrifuged (1000 rpm, 10 min) and the resulting pellet was resuspended in Dulbecco's modified Eagle's medium (DMEM) with 10% non-heat-inactivated fetal bovine serum (FBS) and 1% penicillin–streptomycin (P/S), and used for in vitro PA treatment. 50,000 cells were plated on a 6-well plate overnight in DMEM media with 10%FBS and 1% PS. Further, the cells were pre-treated with vehicle or JWH133 (5 µM) or JWH133 (5 µM) + SR144528 (2 µM) and cells were exposed to PAO1 with 10:1 multiplicity of infection (MOI) for 18 h. The cell culture supernatant was collected and used for ELISA detection of IL-1β and TNF-α (R &D Systems).

### Immunoblot analysis

Lung tissue was lysed in Pierce™ IP Lysis Buffer (ThermoFisher Scientific). The lysates were centrifuged at 12,000 rpm for 15 min at 4 °C. The supernatant was collected and the protein concentration in the supernatant was determined by BCA Protein Assay Kit (Bio-Rad Laboratories). For the immunoblot, 40 µg of the total protein was resolved in SDS-PAGE and electroblotted onto polyvinylidene fluoride (PVDF) membrane. The membrane was blocked with 5% non-fat milk in PBS containing 0.1% Tween-20 for 30 min at room temperature. The membrane was incubated with primary antibodies overnight at 4 °C followed by incubation with secondary antibodies. The membrane was then developed using ECL Western Blot Substrate (ThermoFisher Scientific). The following primary antibodies were used: anti-NLRP3 (1:1000, AG-20B-0014-C100), anti-Caspase-1(p20) (1:1000, AG-20B-0042-C100), anti-Asc (1:500, AG-25B-0006-C100) from Adipogen Life Sciences, Anti-p65 (8242, 1:1000), anti-P-p65-Ser536 (3033, 1:500) from Cell Signaling Technology Inc., and anti-β-actin (1:5000, A5441, Sigma-Aldrich). For the immunoblot analysis of BALF samples, equal volumes of BALF from all groups were loaded. The following primary antibodies were used: anti-Histone H2B (1:500, ab52484, Abcam), anti-CitH3 (1:500, ab5103, Abcam),

### Immunostaining

Paraffin-embedded lung sections (5 µm) were deparaffinized, hydrated, and subjected to antigen retrieval. The sections were stained for myeloperoxidase (MPO) using an anti-MPO antibody (1:200, 79623, Cell Signaling Technology) and for T1α using anti-podoplanin (1:100, NBP203955, Novus Biologicals). Sections were then incubated with fluorochrome-labeled species-specific secondary antibodies and the resultant stained sections were imaged using an Olympus IX73 fluorescent microscope. Three to six mice from each group were used in this experiment.

### Statistical analysis

All results are presented as the mean ± standard error of the mean (SEM). Statistical comparisons were made using Prism 9 (GraphPad Inc). The group differences were analyzed using Student’s t-test or one-way ANOVA followed by a Tukey’s post-hoc multiple comparison tests when appropriate. Differences were considered statistically significant at P < 0.05.

## Results

### CB2R activation alleviates PA-induced alveolar damage and decline in lung function and improves bacterial clearance

**Fig. 1:**
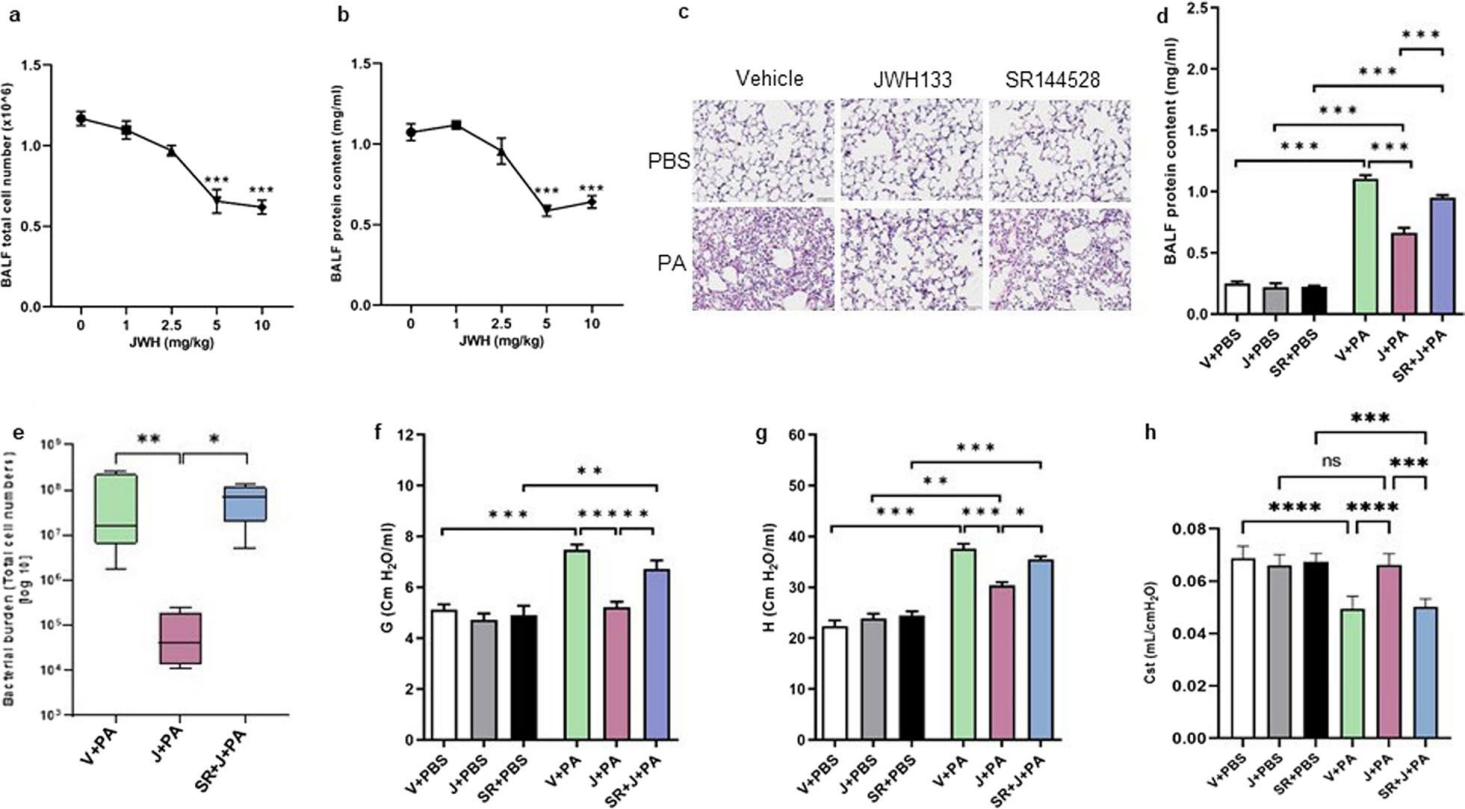
Pharmacological activation of CB2R ameliorates PA-induced alveolar damage and decline in lung function. C57BL/6 J WT mice were treated with vehicle (V) or JWH133 (J) or SR144528 (SR) + JWH133 (J) and exposed to either PBS (control) or PA. Lung injury was assessed after 24 h. Total cell number (**a**) and total protein content (**b**) in the BALF were determined from the mice treated with varying doses of JWH133 and exposed to PA infection (0 mg/kg JWH133:vehicle). **c** Representative H&E images of mice lungs. Total protein content in the BALF (**d**) was determined. **e** The lung bacterial burden was evaluated by measuring the bacterial load in the whole lung. **f **Tissue dampening (G), **g** tissue elastance (H), and, **h** static lung compliance (Cst) were examined using the FlexiVent system. n = 5–8, **p < 0.01, ***p < 0.001, ****p < 0.0001. Data are presented as mean ± SEM

In order to activate CB2R, JWH133 was administered to the mice via the i.p. route, 1 h prior to PA infection. Varying doses (1, 2.5, 5, and 10 mg/kg body weight) of JWH133 were administered to mice to determine its optimum dose. BALF total cell number and total protein content were determined at 24 h, post-PA infection. A dose of 5 mg/kg of JWH133 was found to be highly effective in reducing BALF total cell number (Fig. 1a) and BALF protein content (Fig. 1b) and was continued in further experiments. The effect of CB2R activation in PA-induced lung injury was assessed using lung histology, biochemical indicators, and lung mechanics. At 24 h, after PA instillation, mice displayed typical lung injury and immune cell infiltration. Histological examination of H&E-stained lung sections revealed significant disruption of the alveolar structure, increased inflammatory cell infiltration and protein, and thickened alveolar septae in vehicle-treated mice after i.t. instillation of PA. Pre-treatment of mice with JWH133 reduced these histopathological changes (Fig. 1c). JWH133-treated mice also exhibited reduced BALF protein contents, a marker for lung microvascular permeability (Fig. 1d). In a separate set of experiments, we examined the lung bacterial burden by measuring the bacterial load in the whole lung. Mice that had received JWH133 treatment showed a significantly lower bacterial load in the lung as compared to that in vehicle-treated mice, whereas pharmacological blockade of CB2R by SR144528 led to an increase in the lung bacterial burden (Fig. 1e). Further examination of lung mechanics, using FlexiVent rodent ventilator and FlexiWare software, revealed an increase in parenchymal resistance (G), parenchymal elastance (H), and decrease in static lung compliance (Cst) in response to PA infection. In contrast, JWH133 treatment significantly reduced PA-induced decline in lung function (Fig. 1f, g, h). Pharmacological blockade with an antagonist, SR144528, reversed the effect of JWH133 in the above findings (Fig. 1c–h). These results clearly demonstrate that activation of CB2R offers protection against PA-induced acute lung injury indicators along with the lowering of bacterial burden.

### JWH133 treatment reduces PA-induced lung inflammation

**Fig. 2:**
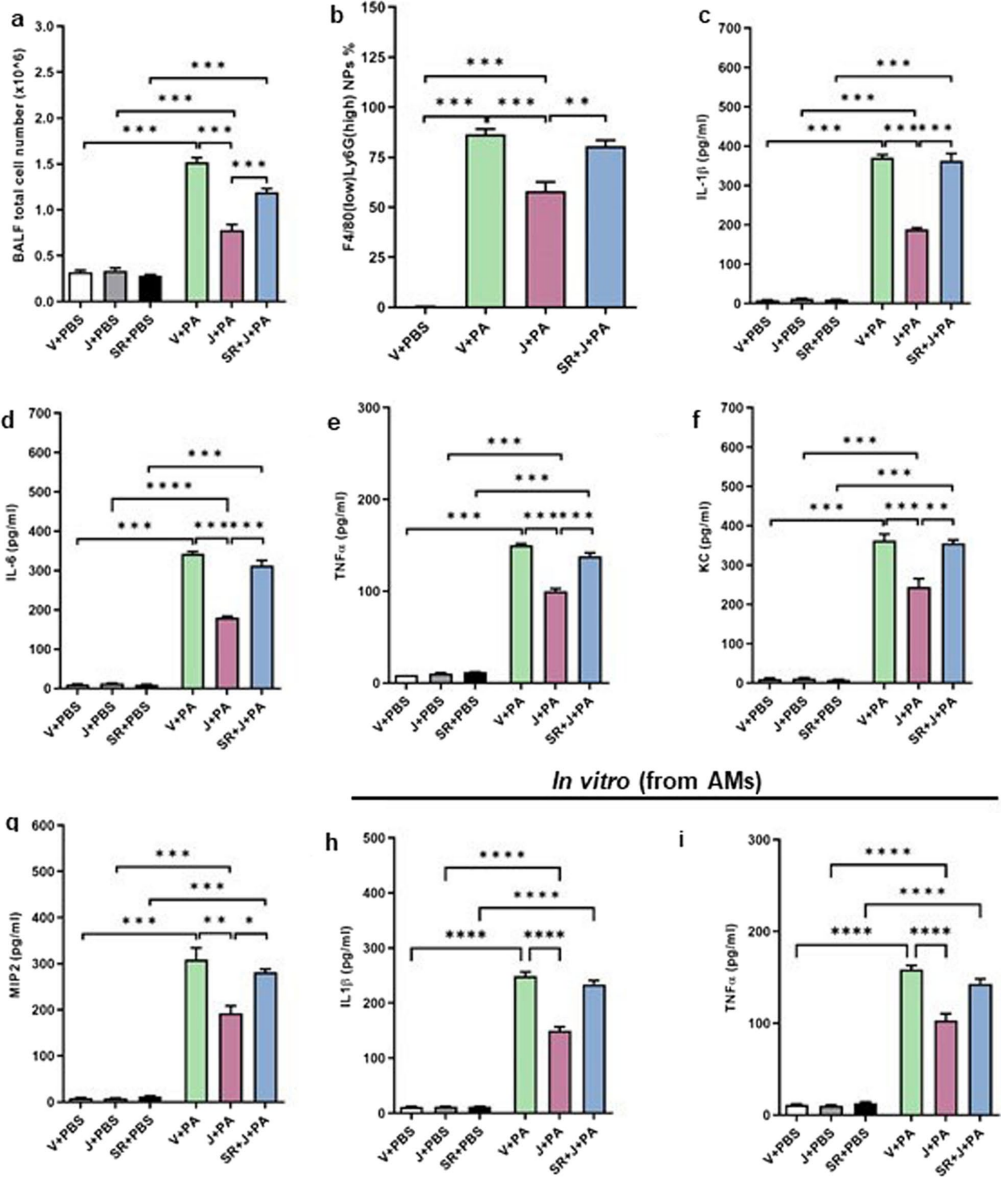
JWH133 treatment reduces PA-induced lung inflammation in mice. C57BL/6 J WT mice were treated with vehicle or JWH133 or SR144528 + JWH133 and exposed to either PBS (control) or PA. BALF was collected after 24 h and total cell number (**a**), and neutrophil population (**b**) were determined. The levels of inflammatory cytokines IL-1β (**c**), IL-6 (**d**), TNF-α (**e**), and chemokines KC (**f**) and MIP-2(**g**) were tested. Mouse primary AMs were exposed to PA and the levels of IL-1β (**h**) and TNF-α (**i**) in the cell culture supernatant were evaluated after 16 h of PA exposure. n = 5–8, **p < 0.01, ***p < 0.001, ****p < 0.0001. Data are presented as mean ± SEM

The infiltration of immune cells into the lung was assessed using the BALF total cell number. CB2R activation was found to significantly reduce the immune cell infiltration into the lungs, as indicated by lower BALF total cell number in JWH133-treated mice as compared to that in vehicle-treated mice (Fig. 2a). The neutrophil population was identified by flow cytometry. Flow cytometry analysis of BALF cells indicated that CB2R activation significantly reduced the percentage of F4/80 (low) Ly6G (high) neutrophils (Fig. 2b). Pre-treatment with antagonist SR144528 resulted in an increase in both total cell numbers and neutrophils (Fig. 2a, b). Pro-inflammatory cytokines are the potent mediators in the pathogenesis of acute lung injury and the balance of pro- to anti-inflammatory cytokines has been shown to be critically important in lung inflammation/resolution. Here, we observed that PA pneumonia led to a significant increase in the levels of major pro-inflammatory cytokines, IL-1β (Fig. 2c), IL-6 (Fig. 2d), and TNF-α (Fig. 2e) in the BALF. Similarly, the BALF levels of chemokines, KC (Fig. 2f), and MIP-2 (Fig. 2g) were also elevated at 24 h, after PA exposure. Interestingly, JWH133-treated mice had significantly lower pro-inflammatory cytokines (Fig. 2c–e) and chemokine levels (Fig. 2f, g). Pre-treatment with SR144528 abrogated the effect of JWH133 (Fig. 2c–g) partially or completely, indicating that anti-inflammatory response was dependent on CB2R activation.

In an in vitro model, the mouse primary AMs were isolated and exposed to PA. The levels of IL-1β and TNF-α in the cell culture supernatant were measured here. JWH133 treatment was found to significantly lower the levels of IL-1β (Fig. 2h) and TNF-α (Fig. 2i). On the contrary, treatment with SR144528 reversed the effect of JWH133 (Fig. 2h, i). The overall findings suggest that CB2R activation significantly reduced PA-induced lung inflammation.

### CB2R deficiency exacerbates PA-induced lung injury and inflammation

**Fig. 3:**
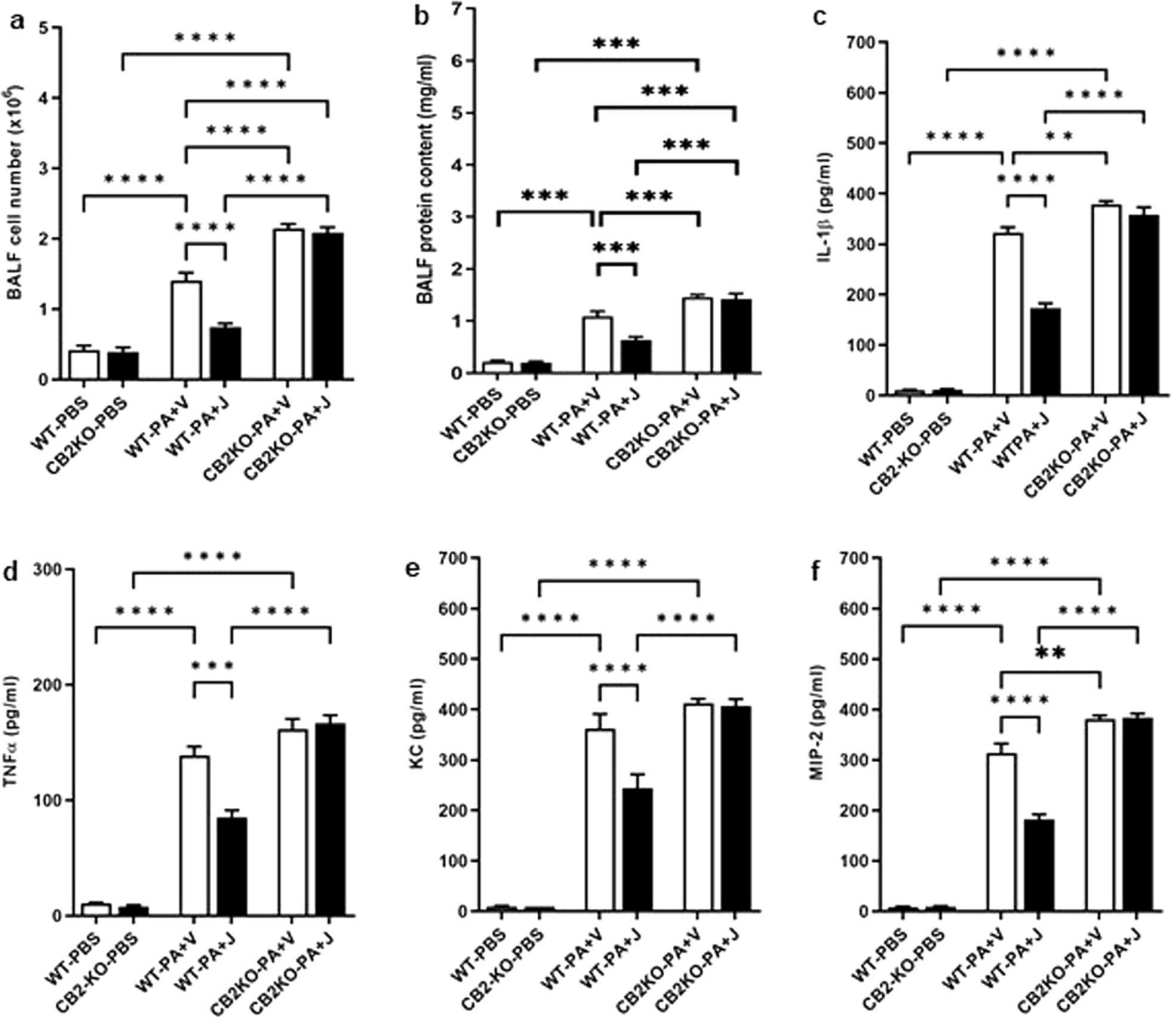
Genetic deletion of CB2R worsens PA-induced lung injury and inflammation. CB2KO and WT mice were pre-treated with JWH133 and exposed PA. Control mice received i.t. injections of PBS. BALF was collected after 24 h and total cell number (**a**), and total protein content (**b**) were determined. ELISA detection of inflammatory cytokines IL-1β (**c**), TNF-α (**d**), and chemokines KC (**e**) and MIP-2 (**f**) in the BALF at 24 h after PA exposure. n = 5-8, **p < 0.01, ***p < 0.001, ****p < 0.0001. Data are presented as mean ± SEM

In order to further confirm the specificity of CB2R in regulating PA-induced lung injury, we used global CB2KO mice. The absence of CB2R increased immune cell infiltration, as revealed by elevated BALF cell numbers in CB2KO mice as compared to those in WT, after PA exposure (Fig. 3a). Further, CB2KO mice had higher BALF protein content, a marker for injury in the alveolar epithelial lining (Fig. 3b). Lung inflammation in WT and CB2KO mice were compared with respect to the levels of inflammatory cytokines and chemokines in their BALF. The levels of major pro-inflammatory cytokines, IL-1β (Fig. 3c) and TNF-α (Fig. 3d) were significantly elevated in the BALF of CB2KO mice as compared to those in WT mice, at 24 h post PA exposure. Similarly, CB2KO mice, when compared to WT mice, had significantly higher levels of BALF chemokines (KC, Fig. 3e; MIP-2, Fig. 3f) after PA infection. JWH133 treatment failed to suppress this PA-induced inflammation in CB2KO mice. On the other hand, when compared to CB2KO mice, WT mice had lower levels of BALF total cell number and total protein content (Fig. 3a, b); and lower levels of pro-inflammatory cytokines, IL-1β and TNF-α (Fig. 3c, d); and chemokines, KC and MIP-2 (Fig. 3e, f). Further, JWH133 treatment significantly reduced these inflammatory markers in WT mice (Fig. 3a–f). These results clearly demonstrate that genetic deletion of CB2R worsened PA-induced lung injury and inflammation.

### Pharmacological activation of CB2R attenuates and genetic deletion of CB2R enhances PA-induced neutrophil activation

**Fig. 4:**
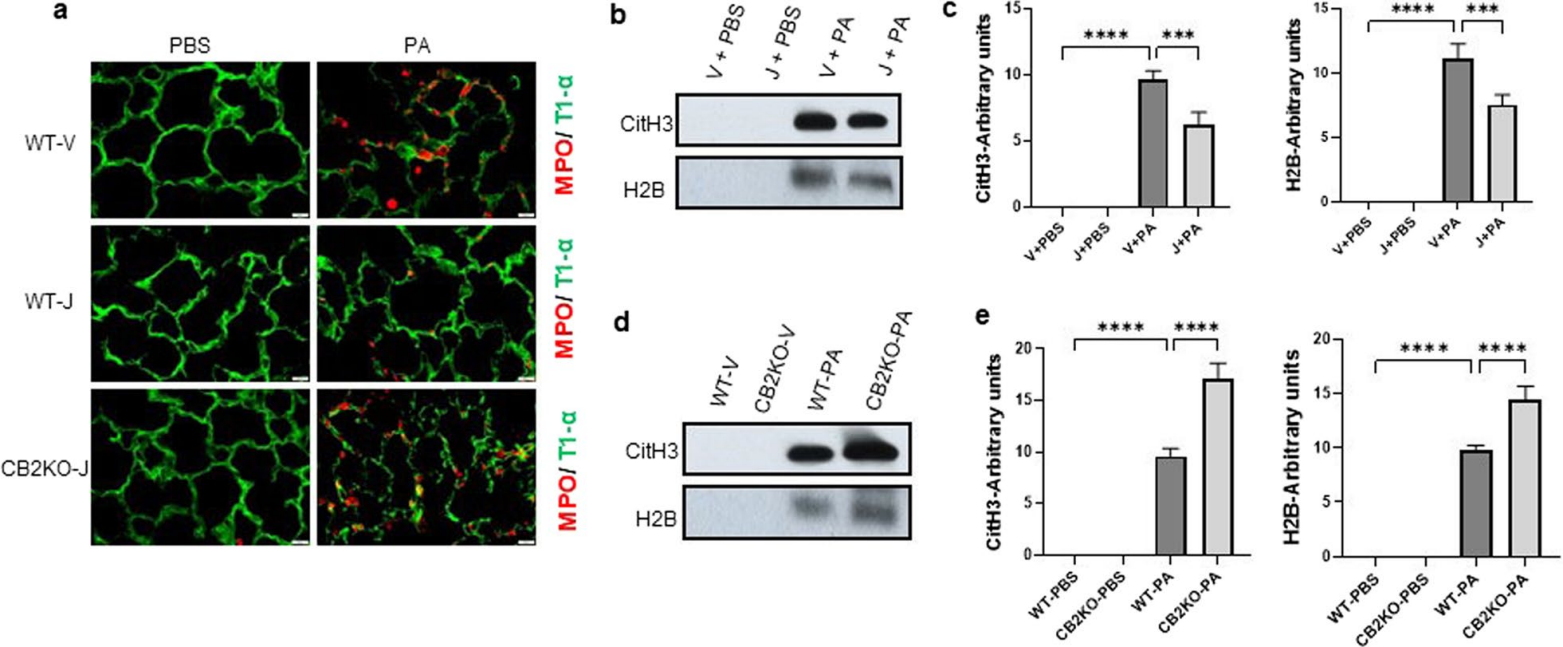
CB2R regulates PA-induced neutrophil activation. **a** Representative Immunostaining image of MPO (red) in WT mice lung pre-treated with vehicle (WT-V) or JWH133 (WT-J) and CB2KO mice lung pre-treated with JWH133 (CB2KO-J) and exposed to PBS (control) or PA for 24 h. T1α staining (green) was used to indicate the epithelial cells. BALF was collected at 24 h after PA infection and the release of CitH3 and H2B were analyzed by immunoblot. Representative immunoblot (**b**) and quantification (**c**) of CitH3 and H2B in BALF from WT mice pretreated with vehicle or JWH133 and exposed to PBS (control) or PA. Representative immunoblot (**d**) and quantification (**e**) of CitH3 and H2B in BALF from WT Vs CB2KO mice pretreated with vehicle or JWH133 and exposed to PBS (control) or PA. n = 4, **p < 0.01***p < 0.001, ****p < 0.0001. Data are presented as mean ± SEM

Our earlier findings have shown that CB2R activation significantly blunted the neutrophil infiltration which was elevated as a result of PA infection (Fig. 2b). In order to verify neutrophil activation, the lung sections were subjected to MPO immunostaining (Fig. 4a). MPO staining in the mice lung, that received vehicle treatment, showed wide distribution (signifying neutrophil infiltration after PA infection), whereas JWH133-treated mice had reduced expressions of MPO in the lung. On the other hand, CB2KO mice showed increased expression of MPO in the lung and JWH133 did not lower or affect the MPO expression (Fig. 4a).

Furthermore, the accumulation of extracellular histones, Citrulline-H3 and Histone-H2B, in the BALF was investigated using immunoblot analysis to understand the release of neutrophil extracellular traps (NETs). BALF Citrulline-H3 and Histone-H2B levels were found to be elevated after PA infection. Mice that had received JWH133 treatment had lower levels of BALF Citrulline-H3 and Histone-H2B (Fig. 4b, c). PA infection enhanced the release of Citrulline-H3 and Histone-H2B in the BALF of CB2KO mice as compared to that in WT mice, and JWH133 failed to conquer these releases in CB2KO mice (Fig. 4d, e). Altogether, these results demonstrate that CB2R activation diminished the excess activation of neutrophils, whereas mice lacking CB2R had elevated neutrophil activation.

### Pharmacological activation of CB2R inhibits and genetic deletion of CB2R enhances NLRP3 inflammasome activation in PA pneumonia

**Fig. 5:**
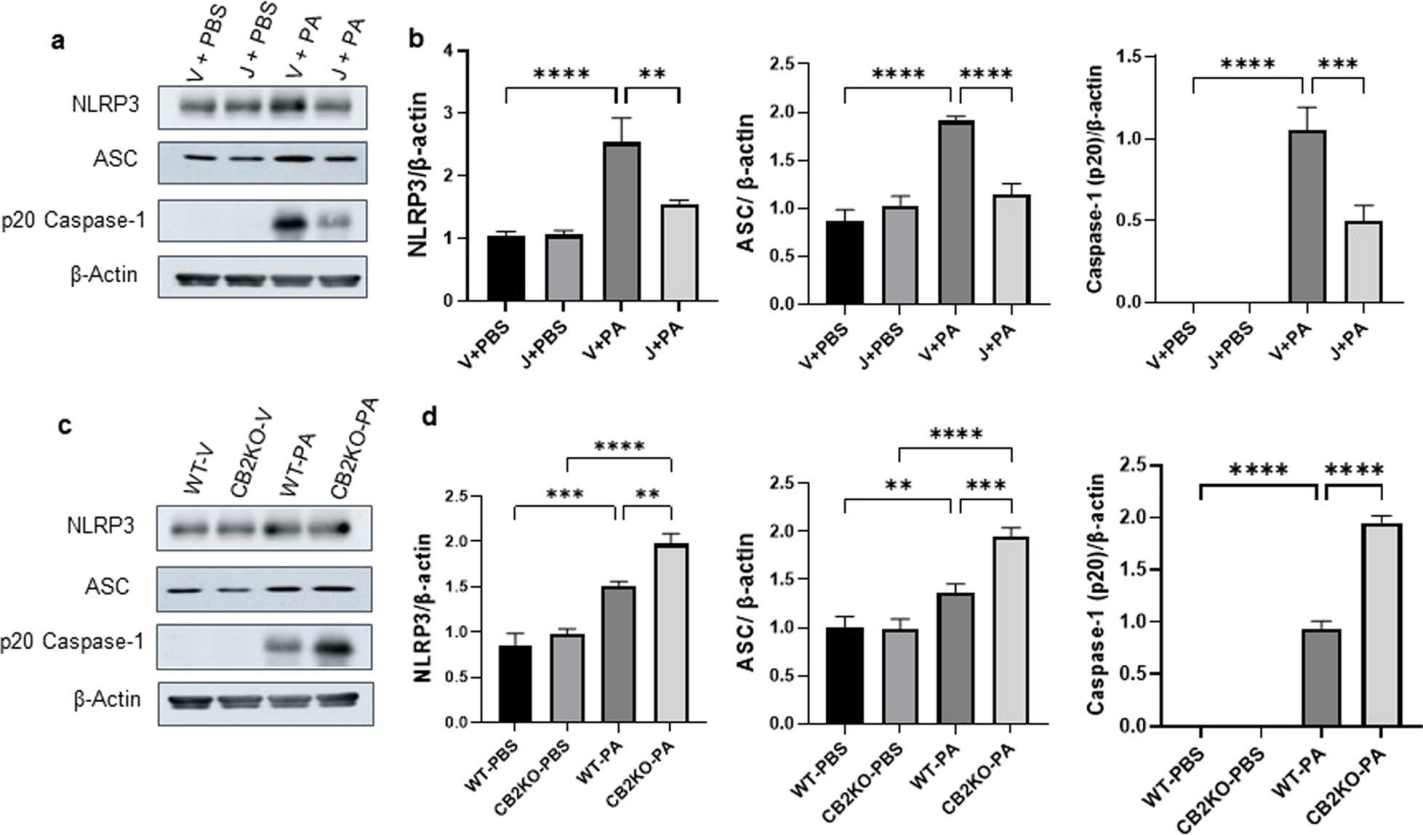
CB2R regulates NLRP3 inflammasome activation in PA pneumonia. C57BL/6 J WT mice received either vehicle (V) or JWH133 (J) and were exposed to PBS or PA. Lung NLRP3 inflammasome activation was assessed by measuring the protein levels of NLRP3, ASC, and (p20) caspase-1. Representative immunoblot images are shown in panel (**a**) and densitometry data are presented in panel (**b**). CB2KO and WT mice received JWH133 and were exposed to PBS or PA and the protein levels of NLRP3, ASC, and (p20) caspase-1 were analyzed by immunoblot. Representative immunoblot images are shown in panel (**c**) and densitometry data are presented in panel (**d**). n = 5, **p < 0.01***p < 0.001, ****p < 0.0001. Data are presented as mean ± SEM

Accumulated pieces of evidence have revealed that the NLRP3 inflammasome pathways are essential for the development of ALI in animal models. In order to investigate the potential of CB2R signaling in regulating the NLRP3 activation, we measured the expression of NLRP3, ASC protein levels, and caspase-1 activation in mice lungs exposed to PA. Our immunoblot results revealed that PA-induced NLRP3 activation was significantly reduced in JWH133-treated mice lungs as compared to those in vehicle-treated mice lungs (Fig. 5a, b). NLRP3, ASC, and Caspase-1(p20) levels were significantly lowered when mice received i.p. injections of JWH133 (Fig. 5a, b). The expressions of NLRP3, ASC, and caspase-1(p20) were enhanced in the lungs of CB2KO mice as compared to those in WT mice, and JWH133 failed to reduce these protein levels in CB2KO mice lungs (Fig. 5c, d). Our earlier findings showed that JWH133-treated mice had significantly lower BALF levels of IL-1β (Fig. 2c), whilst the level of IL-1β was significantly higher in the BALF of CB2KO mice post-PA infection (Fig. 3d). These results support the notion that pharmacological activation of CB2R protects against PA-induced acute lung injury via suppressing NLRP3 inflammasome, whereas elevated NLRP3 inflammasome in CB2KO mice worsens the inflammation and injury at 24 h after PA infection.

### CB2R activation by JWH133 suppresses PA-induced NF-κB activation

**Fig. 6:**
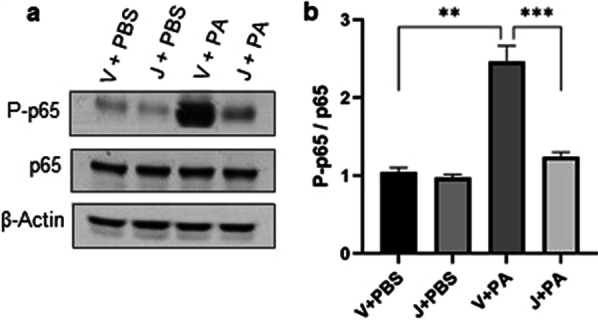
CB2R activation reduces NF-κB activation in PA pneumonia: C57BL/6 J WT mice received either vehicle (V) or JWH133 (J) and were exposed to PBS (control) or PA. NF-κB activation was assessed by measuring the protein levels of P-p65 and p65 in the lung. Representative immunoblot images are shown in panel (**a**) and densitometry data are presented in panel (**b**). n = 5, **p < 0.01***p < 0.001. Data are presented as mean ± SEM

NF-κB is one of the major components mediating the inflammatory process and is a major transcriptional activator of proinflammatory cytokines. Also, the priming signal of NLRP3 inflammasome activation is driven by NF-κB activation. Therefore, it is equally important to understand the effect of CB2R activation by JWH133 in regulating the PA-induced NF-κB activation. Immunoblot analysis revealed an increase in P-p65 expression, an indicator of NF-κB activation, following the PA exposure to mice. However, the JWH133, treatment significantly reduced the P-p65 levels (Fig. 6a, b). These results indicate that CB2R activation restrained the PA-induced expression of inflammatory cytokines by downregulating the activation of the NF-κB and NLRP3 signaling pathways.

## Discussion

In this study, it was found that pharmacological activation of CB2R by a selective synthetic agonist JWH133 significantly reduced PA-induced acute lung injury and inflammation, whereas pre-treatment with antagonist SR144528 abrogated the JWH133 effect confirming the specificity of CB2R. PA-induced lung injury and inflammation were exacerbated in CB2KO mice. CB2R activation reduced the NF-κB and NLRP3 inflammasome activation, whilst genetic deletion of CB2R enhanced the NLRP3 activation post-PA infection.

*Pseudomonas aeruginosa* is a Gram-negative bacillus and an opportunistic pathogen that is one of the main causative agents of hospital-acquired acute lower respiratory tract infections, and a predominant pathogen of ARDS [7, 38]. PA affects a significant number of immunocompromised, critically ill patients [34]. The further acquisition of resistance towards an increasing number of drugs in an already multidrug-resistant PA makes antibiotic therapy ineffective [39] and creates an urgent need for alternative therapies. *P. aeruginosa* strain PA01 was used in this study and i.t. administration of 3 × 10^7^ CFU of PA01 produced quantifiable and reproducible lung injury in mice at 24 h as described in our earlier studies [40].

The endocannabinoid system is widely distributed in mammals and enhancers or inhibitors of endocannabinoid signaling have impacts on numerous physiological and pathological processes, including potentially rewarding anti-inflammatory effects [41]. CB2 receptors, which are predominantly expressed in the peripheral system, represent a promising therapeutic target for various forms of tissue injury and inflammatory diseases [11, 12]. Notably, activation of CB2R is devoid of adverse psychotic effects that can accompany CB1R (expressed mainly in the brain)-based therapies [42]. Hence, the development and use of selective CB2R agonists are of great interest. Amongst the various ligands tested on the basis of off-target activity, selectivity, balance signaling, and, pharmacokinetic profile, JWH133 was found to display the most suitable agonist functions [43]. Hence, JWH133 was used in this study to activate CB2R. An i.p. administration of JWH133 was found to significantly reduce PA-induced immune cell infiltration and BALF protein content (a marker for lung microvascular permeability). Among the various doses tested, the JWH133 dose of 5 mg/kg was found to be effective in reducing BALF total cell number and total protein content. The dose of 5 mg/kg of JWH133 was used throughout our study.

The lung damage and reduced lung function observed at 24 h after PA infection was significantly reversed by CB2R activation. Notably, mice that received JWH133 treatment had significantly lower lung bacterial load and pharmacological blockade of CB2R abrogated the JWH133-mediated effect. PA is known to induce the expression of pro-inflammatory mediators that modulate inflammatory pathways and further exacerbate lung inflammation [44]. In our model, intratracheal administration of PA led to an increase in inflammatory cytokines IL-1β, TNF-α, and IL-6 and chemokines KC and MIP2 (functional homologues of IL-8) in vehicle-treated mice. However, mice that had received JWH133 treatment showed significantly lower levels of IL-1β, TNF-α, and IL-6, and chemokines, KC and MIP2. In the case of CB2KO mice, an increase in BALF total cell number was seen, indicating an influx of immune cells into the lung. The co-occurrence of increased immune cell infiltration and increased production of inflammatory cytokines and chemokines suggests that CB2KO mice are more susceptible to PA-induced respiratory infection. Treatment with JWH133 was also found to be futile in suppressing PA-induced injury and inflammatory indicators in CB2KO mice, further substantiating the specificity of CB2R signaling in protection against PA pneumonia. All these findings suggest that CB2R plays a key role in regulating the inflammatory response to PA01 in an acute respiratory infection condition by preventing over-exaggerated inflammation.

The persistence of a pathogen like PA leads to elevated immune responses and severe cytotoxicity. Neutrophils are among the first immune cells recruited to the inflammation site and their activation is characterized by neutrophil extracellular trap (NET) formation (NETosis) [45] and myeloperoxidase release from lysosomes [46]. Excessive activation of neutrophils or their prolonged survival in tissues during inflammation is associated with high tissue damage. Previous studies have reported that type I interferons (IFNs)-mediated excessive activation of neutrophils enhances the NETosis, which in turn increases the PA bacterial load in the lung exacerbating the inflammation [47]. The CB2 receptor has been shown to modulate immune cell functions, both in in vitro and in animal models of inflammatory diseases, with its absence enhancing the neutrophil recruitments into the spleen as seen in a mouse model of endotoxemia [48]. CB2R has also been found to suppress neutrophil recruitment to the dorsal air pouch in a murine dorsal air pouch inflammation model [49]. In the current study, we found that CB2R activation reduced the PA-induced neutrophil infiltration, as revealed by a lower percentage of neutrophils in the BALF of mice treated with JWH133. Further, pharmacological activation of CB2R was found to reduce the levels of MPO in the lung tissues, and the levels of H2B and CitH3 in the BALF, markers of NETosis. Our data indicate that a decrease in over-exuberant neutrophil recruitment by CB2R activation could lead to a novel therapeutic target for PA-induced lung injury. However, further studies are needed to determine the exact pathway and the related cellular key players that are crucial for CB2R mediated effect on neutrophil functions.

Inflammasomes are activated during pathogen invasion, and sterile inflammation and tissue injury, leading to cell death. Cell death can also result in the secretion of another round of inflammasome activators, like uric acid and ATP, which further activate inflammasomes in a paracrine manner [50]. Among the inflammasomes, NLRP3 has emerged as one of the most promising therapeutic targets in translational immunology [26]. The NLRP3 inflammasome activation includes a priming signal that comes from the stimulation of pathogen recognition receptors (PRRs), which further activates NF-κB and mitogen-activated protein kinase (MAPK). The elements such as ATP, pore formation, potassium efflux, lysosomal rupture, and reactive oxygen contribute to enhancing the NLRP3 activation. [29, 51]. The involvement of NLRP3 activation in lung injury and inflammation has also been highlighted in a number of studies. Jones et al. have reported the critical role of the NLRP3 inflammasome in the development of hypoxemia in LPS/mechanical ventilation lung injury [52]. Another study has also shown the contribution of NLRP3 activation toward mechanical stretch-induced lung inflammation-injury and LPS-induced acute lung injury [53]. A recent study has also demonstrated that PA infection results in the activation of NLRP3 inflammasome in mice lungs, as indicated by induction of NLRP3 and ASC as well as caspase-1 and IL-1β. This study further demonstrated that chemical inhibition of equilibrative nucleoside transporter (ENT) and genetic deletion of ENT1 inhibited the NLRP3 inflammasome activation and protected against PA-induced acute lung injury [37].

Studies on cannabinoids have shown their inhibitory role in NLRP3 inflammasome activation and highlighted their potent anti-inflammatory effects [31]. Other studies have also reported that activating CB2R ameliorates the pathogenesis of experimental autoimmune encephalomyelitis by suppressing the NLRP3 inflammasome [54] and the protective effect of colitis gained via CB2R-mediated inhibition of NLRP3 inflammasome [55].

The aforementioned findings provide a good background towards discussing the role of CB2R activation in regulating NLRP3 inflammasome in our mice model of *P. aeruginosa* pneumonia. Here, we observed that intratracheal administration of PA led to an activation of the NLRP3 inflammasome, as indicated by induction of NLRP3, activation of caspase-1, and release of IL-1β. This is in accordance with previous studies that underlined the importance of IL-1β and IL-18 in mediating the PA-induced acute lung injury and activation of NLRP3 inflammasome [29, 30]. Interestingly, our study demonstrated that pharmacological activation of CB2R suppressed the activation of inflammasomes. Mice that received JWH133 treatment had significantly lower levels of NLRP3, ASC, and reduced caspase-1 activation at 24 h after PA infection. The diminished levels of the active caspase-1 also reduce the production of IL-1β from its inactive precursor form. Our results showed that IL-1β levels were significantly reduced in the BALF of JWH133-treated mice. Similarly, JWH133 treatment resulted in reduced IL-1β release from mouse primary AMs in response to PA infection as observed by lower IL-1β levels in the supernatant of AMs treated with JWH133 as compared to that of vehicle-treated AMs. Furthermore, genetic deletion of CB2R amplified the NLRP3 activation post-PA infection, besides the observation of higher levels of NLRP3, ASC, and caspase-1(p20) levels in the lungs of CB2KO mice. Our results have also demonstrated that PA-induced NF-κB activation (primary regulator of inflammation in ALI and enhancer of NLRP3 activation) was significantly reduced by pharmacological activation of CB2R. Notably, JWH133 treatment reduced both NF-κB and NLRP3 activation. All these findings lead us to speculate that inhibition of NF-κB /NLRP3 inflammasome is the most plausible mechanism through which CB2R ameliorates the PA-induced acute lung injury and inflammation.

## Conclusion

In summary, our findings have uncovered the role of CB2R in modulating PA-induced acute lung injury and inflammation in mice. We have also demonstrated that pharmacological activation of CB2R by a selective synthetic agonist JWH133 promotes the resolution of PA-induced lung inflammation and injury, whereas genetic deletion of CB2R aggravates the injury. Our findings also show that NLRP3 inflammasome signaling is involved in mediating the protective effect of CB2R. These results add further evidence to strongly suggest that targeting CB2R could lead to novel strategies for the prevention of PA pneumonia and subsequent ARDS.

## Data Availability

Data supporting the findings of this study are available from the corresponding author upon request.

## Abbreviations

ALI: Acute lung injury
ARDS: Acute respiratory distress syndrome
PA: *Pseudomonas aeruginosa*
MDR: Multidrug-resistant
VAP: Ventilator associated-pneumonia
CB2R: Cannabinoid-2 receptor
GPCR: G-protein coupled receptor
NLRP3: NOD-leucine-rich repeat
LRR: Pyrin domain-containing protein 3
ASC: Apoptosis-associated speck-like protein containing a caspase recruitment domain
DAMP: Damage-associated molecular patterns
PAMP: Pathogen-associated molecular patterns
PVDF: Polyvinylidene fluoride
BALF: Bronchoalveolar lavage fluid
AM: Alveolar macrophages
DMEM: Dulbecco's modified Eagle's medium
P/S: Penicillin–streptomycin
MPO: Myeloperoxidase
WT: Wild type
KO: Knock out
i.t: Intratracheal
i.p.: Intraperitoneal
SEM: Standard error of the mean
CitH3: Citrulline-H3

## References

[1] Bauer TT, Ewig S, Rodloff AC, Müller EE (2006). Acute respiratory distress syndrome and pneumonia: a comprehensive review of clinical data. Clin Infect Dis.

[2] Fan E, Brodie E, Slutsky AS (2018). Acute respiratory distress syndrome advances in diagnosis and treatment. JAMA.

[3] Matthay MA, Zemans RL, Zimmerman GA, Arabi YM, Beitler JR, Mercat A, Herridge M, Randolph AG, Calfee CS (2019). Acute respiratory distress syndrome. Nat Rev Dis Primers.

[4] Liu KD (2017). Acute respiratory distress syndrome. N Engl J Med.

[5] Bassetti M, Vena A, Croxatto A, Righi E, Guery B. How to manage *Pseudomonas aeruginosa* infections. Drugs Context. 2018; (7) 212527. 10.7573/dic.212527 PMC5978525 29872449

[6] Ramirez-Estrada S, Borgatta B, Rello J (2016). *Pseudomonas aeruginosa* ventilator-associated pneumonia management. Infect Drug Resist.

[7] Jones RN (2010). Microbial etiologies of hospital-acquired bacterial pneumonia and ventilator-associated bacterial pneumonia. Clin Infect Dis.

[8] Tacconelli E, Magrini N (2017). Global priority list of antibiotic-resistant bacteria to guide research, discovery, and development of new antibiotics.

[9] Lu HC, Mackie K (2016). An introduction to the endogenous cannabinoid system. Biol Psychiatry.

[10] Chiurchiu V, Battistini L, Maccarrone M (2015). Endocannabinoid signaling in innate and adaptive immunity. Immunology.

[11] Pacher P, Batkai S, Kunos G (2006). The endocannabinoid system as an emerging target of pharmacotherapy. Pharmacol Rev..

[12] Marzo V (2018). New approaches and challenges to targeting the endocannabinoid system. Nat Rev Drug Discov.

[13] Cristino L, Bisogno T, Di Marzo V (2019). Cannabinoids and the expanded endocannabinoid system in neurological disorders. Nat Rev Neurol.

[14] Pacher P, Kunos G (2013). Modulating the endocannabinoid system in human health and disease—successes and failures. FEBS J.

[15] Zou S, Kumar U (2018). Cannabinoid receptors and the endocannabinoid system: signaling and function in the central nervous system. Int J Mol Sci.

[16] Basu S, Dittel BN (2011). Unraveling the complexities of cannabinoid receptor 2 (CB2) immune regulation in health and disease. Immunol Res.

[17] Turcotte C, Blanchet MR, Laviolette M, Fla-mand N (2016). The CB2 receptor and its role as a regulator of inflammation. Cell Mol Life Sci.

[18] Mukhopadhyay P, Baggelaar M, Erdelyi K, Cao Z, Cinar R, Fezza F, Ignatowska-Janlowska B, Wilkerson J, van Gils N, Hansen T, Ruben M, Soethoudt M, Heitman L, Kunos G, Maccarrone M, Lichtman A, Pacher P, Van der Stelt M (2016). The novel, orally available and peripherally restricted selective cannabinoid CB2 receptor agonist LEI-101 prevents cisplatin-induced nephrotoxicity. Br J Pharmacol.

[19] Mukhopadhyay P, Rajesh M, Pan H, Patel V, Mukhopadhyay B, Bátkai S, Gao B, Haskó G, Pacher P (2010). Cannabinoid-2 receptor limits inflammation, oxidative/nitrosative stress, and cell death in nephropathy. Free Radic Biol Med.

[20] Bátkai S, Osei-Hyiaman D, Pan H, El-Assal O, Rajesh M, Mukhopadhyay P, Hong F, Harvey-White J, Jafri A, Haskó G, Huffman JW, Gao B, Kunos G, Pacher P (2007). Cannabinoid-2 receptor mediates protection against hepatic ischemia/reperfusion injury. FASEB J.

[21] Rajesh M, Pan H, Mukhopadhyay P, Bátkai S, Osei-Hyiaman D, Haskó G, Liaudet L, Gao B, Pacher P (2007). Cannabinoid-2 receptor agonist HU-308 protects against hepatic ischemia/reperfusion injury by attenuating oxidative stress, inflammatory response, and apoptosis. J Leukoc Biol.

[22] Turcotte C, Blanchet M, Laviolette M, Flamand N (2016). Impact of cannabis, cannabinoids and endocannabinoids in the lungs. Front Pharmacol.

[23] Fu Q, Zheng Y, Dong X, Jiang CG (2017). Activation of cannabinoid receptor type 2 by JWH133 alleviates bleomycin-induced pulmonary fibrosis in mice. Oncotarget.

[24] Franchi L, Muñoz-Planillo R, Núñez G (2012). Sensing and reacting to microbes through the inflammasomes. Nat Immunol.

[25] Strowig T, Henao-Mejia J, Elinav E, Flavell R (2012). Inflammasomes in health and disease. Nature.

[26] Guo H, Callaway JB, Ting JP (2015). Inflammasomes: mechanism of action, role in disease, and therapeutics. Nat Med.

[27] Paik S, Kim JK, Silwal P, Sasakawa C, Jo E (2021). An update on the regulatory mechanisms of NLRP3 inflammasome activation. Cell Mol Immunol.

[28] He Y, Hara H, Núñez G (2016). Mechanism and regulation of NLRP3 inflammasome activation. Trends Biochem Sci.

[29] Rathinam VA, Fitzgerald KA (2016). Inflammasome complexes: emerging mechanisms and effector functions. Cell.

[30] Kumar SR, Paudel S, Ghimire L, Bergeron S, Cai S, Zemans RL, Downey GP, Jeyaseelan S (2018). Emerging roles of inflammasomes in acute pneumonia. Am J Respir Crit Care Med.

[31] Wu Z, Yan Z, Schwartz DE, Yu J, Malik AB, Hu G (2013). Activation of NLRP3 inflammasome in alveolar macrophages contributes to mechanical stretch-induced lung inflammation and injury. J Immunol.

[32] Fukumoto J, Fukumoto I, Parthasarathy PT (2013). NLRP3 deletion protects from hyperoxia-induced acute lung injury. Am J Physiol Cell Physiol.

[33] Jin L, Batra S, Jeyaseelan S (2017). Deletion of Nlrp3 augments survival during polymicrobial sepsis by decreasing autophagy and enhancing phagocytosis. J Immunol.

[34] Schultz MJ, Rijneveld AW, Florquin S, Edwards CK, Dinarello CA, van der Poll T (2002). Role of interleukin-1 in the pulmonary immune response during *Pseudomonas aeruginosa* pneumonia. Am J Physiol Lung Cell Mol Physiol.

[35] Schultz MJ, Knapp S, Florquin S (2003). Interleukin-18 impairs the pulmonary host response to *Pseudomonas aeruginosa*. Infect Immun.

[36] Cohen TS, Prince AS (2013). Activation of inflammasome signaling mediates pathology of acute *P. aeruginosa* pneumonia. J Clin Invest.

[37] Chambers ED, White A, Vang A, Wang Z, Ayala A, Weng T, Blackburn M, Choudhary G, Rounds S, Lu Q (2020). Blockade of equilibrative nucleoside transporter 1/2 protects against *Pseudomonas aeruginosa*-induced acute lung injury and NLRP3 inflammasome activation. FASEB J.

[38] Barbier F, Andremont A, Wolff M, Bouadma L (2013). Hospital-acquired pneumonia and ventilator-associated pneumonia: recent advances in epidemiology and management. Curr Opin Pulm Med.

[39] Sawa T, Shimizu M, Moriyama K, Wiener-Kronish JP (2018). Association between *Pseudomonas aeruginosa* type III secretion, antibiotic resistance, and clinical outcome: a review. Crit Care.

[40] Nagre N, Cong X, Terrazas C, Pepper I, Schreiber JM, Fu H, Sill JM, Christman JW, Satoskar AR, Zhao X (2018). Inhibition of macrophage complement receptor CRIg by TRIM72 polarizes innate immunity of the lung. Am J Respir Cell Mol Biol.

[41] Barrie N, Manolios N (2017). The endocannabinoid system in pain and inflammation: its relevance to rheumatic disease. Eur J Rheumatol.

[42] Dhopeshwarkar A, Mackie K (2014). CB2 cannabinoid receptors as a therapeutic target—what does the future hold?. Mol Pharmacol.

[43] Soethoudt M, Grether U, Fingerle J (2017). Cannabinoid CB_2_ receptor ligand profiling reveals biased signaling and off-target activity. Nat Commun.

[44] Sawa T (2014). The molecular mechanism of acute lung injury caused by *Pseudomonas aeruginosa*: from bacterial pathogenesis to host response. J Intensive Care.

[45] Jorch SK, Kubes P (2017). An emerging role for neutrophil extracellular traps in noninfectious disease. Nat Med.

[46] Metzler KD, Goosmann C, Lubojemska A, Zychlinsky A, Papayannopoulos V (2014). A myeloperoxidase containing complex regulates neutrophil elastase release and actin dynamics during NETosis. Cell Rep.

[47] Pylaeva E, Bordbari S, Spyra I, Decker AS, Häussler S, Vybornov V, Lang S, Jablonska J (2019). Detrimental effect of type I IFNs during acute lung infection with *Pseudomonas aeruginosa* is mediated through the stimulation of neutrophil NETosis. Front Immunol.

[48] Kapellos TS, Recio C, Greaves DR, Iqbal AJ (2017). Cannabinoid receptor 2 modulates neutrophil recruitment in a murine model of endotoxemia. Mediators Inflamm.

[49] Kapellos TS, Hussain MT, Rainger GE, Greaves DR, Iqbal AJ (2019). Cannabinoid Receptor 2 deficiency exacerbates inflammation and neutrophil recruitment. FASEB J.

[50] Broz P, Dixit V (2016). Inflammasomes: mechanism of assembly, regulation and signalling. Nat Rev Immunol.

[51] Kelley N, Jeltema D, Duan Y, He Y (2019). The NLRP3 inflammasome: an overview of mechanisms of activation and regulation. Int J Mol Sci.

[52] Jones HD, Crother TR, Gonzalez-Villalobos RA (2014). The NLRP3 inflammasome is required for the development of hypoxemia in LPS/mechanical ventilation acute lung injury. Am J Respir Cell Mol Biol.

[53] Suryavanshi SV, Kovalchuk I, Kovalchuk O (2021). Cannabinoids as key regulators of inflammasome signaling: a current perspective. Front Immunol.

[54] Shao BZ, Wei W, Ke P, Xu ZQ, Zhou JX, Liu C (2014). Activating cannabinoid receptor 2 alleviates pathogenesis of experimental autoimmune encephalomyelitis via activation of autophagy and inhibiting NLRP3 inflammasome. CNS Neurosci Ther.

[55] Ke P, Shao BZ, Xu ZQ, Wei W, Han BZ, Chen XW, Su DF, Liu C (2016). Activation of Cannabinoid receptor 2 ameliorates DSS-induced colitis through inhibiting NLRP3 inflammasome in macrophages. PLoS ONE.

